# Microplastics in agriculture – a potential novel mechanism for the delivery of human pathogens onto crops

**DOI:** 10.3389/fpls.2023.1152419

**Published:** 2023-08-10

**Authors:** Richard S. Quilliam, Chloe J. Pow, Dativa J. Shilla, James J. Mwesiga, Daniel A. Shilla, Luke Woodford

**Affiliations:** ^1^ Biological and Environmental Sciences, Faculty of Natural Sciences, University of Stirling, Stirling, United Kingdom; ^2^ Department of Chemistry, Dar es Salaam University College of Education (DUCE), Dar es Salaam, Tanzania; ^3^ Department of Aquatic Sciences and Fisheries Technology, University of Dar es Salaam, Dar es Salaam, Tanzania

**Keywords:** human health, irrigation, microplastic-soil-crop interactions, plastic pollution, plastisphere, wastewater

## Abstract

Mulching with plastic sheeting, the use of plastic carriers in seed coatings, and irrigation with wastewater or contaminated surface water have resulted in plastics, and microplastics, becoming ubiquitous in agricultural soils. Once in the environment, plastic surfaces quickly become colonised by microbial biofilm comprised of a diverse microbial community. This so-called ‘plastisphere’ community can also include human pathogens, particularly if the plastic has been exposed to faecal contamination (e.g., from wastewater or organic manures and livestock faeces). The plastisphere is hypothesised to facilitate the survival and dissemination of pathogens, and therefore plastics in agricultural systems could play a significant role in transferring human pathogens to crops, particularly as microplastics adhering to ready to eat crops are difficult to remove by washing. In this paper we critically discuss the pathways for human pathogens associated with microplastics to interact with crop leaves and roots, and the potential for the transfer, adherence, and uptake of human pathogens from the plastisphere to plants. Globally, the concentration of plastics in agricultural soils are increasing, therefore, quantifying the potential for the plastisphere to transfer human pathogens into the food chain needs to be treated as a priority.

## Introduction

Globally, more than 800 million farmers are involved with urban agriculture, and about a quarter of these practice market-oriented farming (Qadir et al., 2010). Urban and peri-urban farmers in low- and middle-income countries (LMICs) can enhance household income by producing perishable crops such as leafy vegetables for sale in local markets (Zezza and Tasciotti, 2010), which is crucial for providing a continual supply of vitamin-rich vegetables to the community. Importantly, such production of fresh vegetables and leafy greens is fundamental for alleviating hidden hunger (the deficiency in micronutrients, vitamins, and minerals in the diet) in urban and peri-urban areas (Chagomoka et al., 2017).

Farmers growing crops in LMICs often use wastewater [in this paper we refer to ‘wastewater’ as the use of raw, partly treated, or diluted wastewater, from predominantly domestic sources] for irrigation as it provides a free source of nitrogen and phosphorus (and thus, less money spent on fertilisers), and can be more reliable or cheaper than other surface water sources. Use of wastewater in urban vegetable farms not only lessens the pressure on water resources but also increases water productivity through reuse of water and nutrients (Qadir et al., 2020; Minhas et al., 2022). Wastewater (either untreated or secondary/tertiary treated wastewater) also makes up a significant proportion of irrigation used in agricultural systems in the USA, Australia, and many other countries with arid and semi-arid regions, e.g., Saudi Arabia, Tunisia, Pakistan. However, wastewater irrigation is often associated with enteric pathogens (Partyka and Bond, 2022) and more recently microplastics (defined as plastic particles < 5 mm), which in the last few decades have become ubiquitous in the environment (Uddin et al., 2020; Pérez-Reverón et al., 2022). Polymers such as polyethylene (PE), polypropylene (PP), and polystyrene (PS) can originate from either a primary source (e.g., from cosmetics, or manufacturing processes), or as a secondary source following the fragmentation of larger pieces of plastic.

The sources and migration pathways of microplastics in more intensive agricultural systems, e.g., in China, have been comprehensively reviewed (Jin et al., 2022; Yu et al., 2022), with the main soil inputs coming from mulching with plastic sheeting, the application of seed coatings containing plastic carriers, and irrigation (Zhou et al., 2021; Pérez-Reverón et al., 2022). Importantly, plastics (and microplastics) in the environment become rapidly colonised by microbial biofilms formed by the microbial secretion of extracellular polymeric substances onto the plastic surface and can provide a novel hydrophobic ecological habitat capable of supporting diverse microbial communities. Such biofilm situated at the interface between the plastic surface and the environment has been termed the ‘plastisphere’ (Zettler et al., 2013), and is hypothesised to provide a protective environment that enables microorganisms to grow in hostile habitats and facilitate their dispersal (Amaral-Zettler et al., 2020). However, the plastisphere can also contain bacterial and viral human pathogens capable of retaining their virulence and infectivity (Moresco et al., 2021; Metcalf et al., 2022; Ormsby et al., 2023), and therefore microplastics have the potential to act as a significant vector of pathogens, particularly if they have been in contact with a source of faecal contamination.

**Figure 1:**
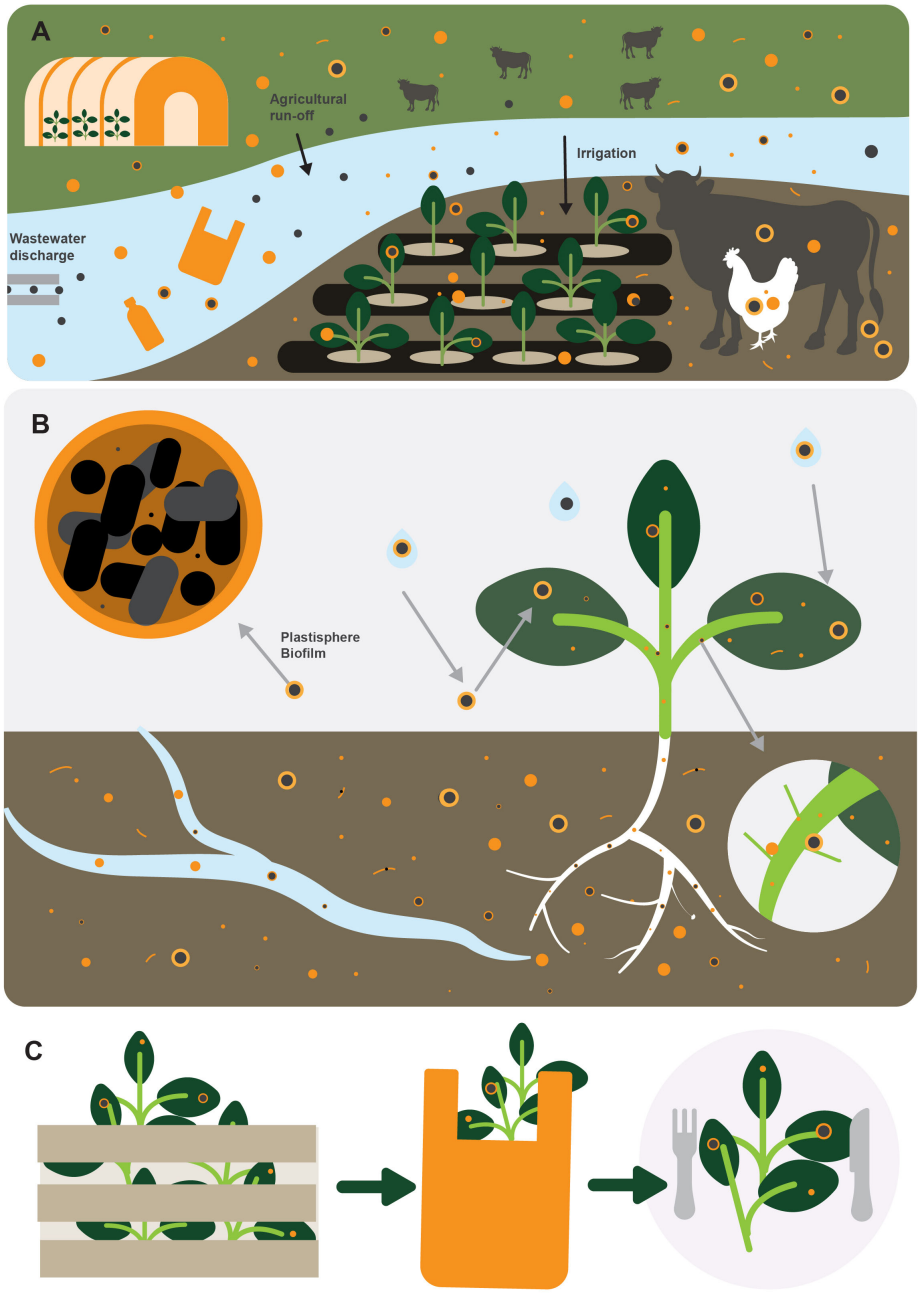
**(A)** plastics and microplastics (orange circles and fibres) can enter agricultural systems from contaminated irrigation water, livestock faeces, and organic fertilisers, or from the breakdown of larger pieces of plastics such as from mulching film or polytunnels. Once in the environment, plastic quickly becomes colonised by microbial biofilm, termed the ‘plastisphere’ (orange circles with black dots), which often contains human pathogens; **(B)** the plastisphere can increase survival and dissemination of human pathogens and could facilitate their transfer to the rhizosphere and the developing roots, or onto the surface of leaves and fruit, either directly through irrigation, or indirectly through splash from the soil; **(C)** once microplastics have adhered to plant tissue it is not known whether human pathogens in the plastisphere could subsequently be transferred to the plant; however, it is difficult to remove microplastics from plant leaves (even by washing), and so they could remain attached during the harvesting and retail processes and thus increase the potential for human pathogens to enter the food chain.

The long-term effects of plastics and microplastics in soil and crop systems are currently undergoing intense research focus (e.g., Li et al., 2022; Zhou et al., 2022; Li et al., 2023). Yet, despite this, the potential risk to human health of growing vegetables in soils containing pathogen-colonised plastics, and further irrigating crops with wastewater contaminated with both faecally associated pathogens and a potentially high load of microplastics, has never before been considered (**Figure 1**). Therefore, the aim of this paper is to explore the potential for human pathogens adhering to plastics and microplastics to enter agricultural systems and be introduced into the food chain.

## Transfer of plastics into agricultural systems: co-pollutant potential for human pathogens to enter the food chain?

An increasing proportion of soils used for crop production are heavily contaminated with plastics (Chen et al., 2020). Globally, the volume of plastic in soils is predicted to increase due to practices such as plastic mulching and seed treatments (Li et al., 2022) together with the effects of rapid urbanisation, and particularly in LMICs, weak infrastructural capacity for waste management (Ferronato and Torretta, 2019). Recently, a meta-analysis by Zhang et al. (2022) has demonstrated that plastic residues in soil can alter nutrient cycling and inhibit plant growth, which is likely to be driven by plastics modifying the functioning and diversity of soil microbial communities, e.g., by potentially stimulating enhanced nutrient and carbon cycling (Bandopadhyay et al., 2018; Xiao et al., 2022). However, in addition to soil type and environmental conditions, the consequences of plastics in soil are determined by a number of other factors including size, shape, concentration, and polymer type (Iqbal et al., 2023). Recently, degradable ‘bio-plastics’ have been suggested as an alternative to plastic mulching, although current reports have indicated variable consequences for crop yield, soil health, and nutrient cycling (Brown et al., 2023; Chu et al., 2023; Reay et al., 2023).

The presence of human pathogens on the surfaces of environmental plastic pollution has been well documented over the last decade (Metcalf et al., 2022) particularly in marine systems (Bowley et al., 2021). Microplastics and human pathogens often come into close contact with each other in aquatic systems, either during transit through wastewater treatments plants (WWTPs) or in contaminated surface waters, e.g., from agricultural run-off, direct defecation from livestock, or from sewage discharge (**Figure 1**). The preference for many bacterial species to colonise the surfaces of plastics (or to attach to the biofilm on the plastic surface) rather than remain planktonic has led to the hypothesis that microplastics can become enriched with human pathogens and increase their persistence and dissemination in the environment (Junaid et al., 2022).

*In-situ* delivery of microplastics colonised by human pathogens could be facilitated by irrigation with wastewater, which is common in LMICs where most vegetables are grown in river-valleys, where in the dry season wastewater constitutes the only available surface water for irrigation (Qadir et al., 2010). More widely, crops are irrigated with surface waters from rivers, streams, and lakes, which are commonly contaminated with treated or untreated wastewater effluent (Shehu et al., 2022). Estimates of global annual inputs of microplastic into the environment includes 10^15^ particles entering the aquatic environment in treated effluent, and a further 10^16^ particles released in untreated effluent (Uddin et al., 2020). Therefore, there is a high likelihood that crops are already being irrigated with water containing microplastics that are colonised with human pathogens (**Figure 1**).

Other routes for microplastics to enter agronomic systems include the use of organic fertilisers, composts, or biosolids (Weithmann et al., 2018; Vithanage et al., 2021). The application of animal manure to fields, or the rotation of grazing livestock, can increase the abundance of microplastics in the soil (Beriot et al., 2021; Yang et al., 2021), which has likely originated from the ingestion by livestock of microplastics in feed, feed bags, and containers (Wu et al., 2021). The high concentrations of potentially zoonotic and human pathogens in livestock faeces (e.g., *E. coli* O157, *Salmonella* spp., *Campylobacter* spp.) provides the opportunity for microplastics being transported through the livestock gut to have already become colonised with pathogens before they enter the soil. Furthermore, microplastics have also been extracted from wildlife faeces including rats, mice, hedgehogs, and voles, increasing the potential loading of contaminated microplastics into agricultural soils (Thrift et al., 2022).

## Can plastics act as a vector for the transfer of human pathogens onto crops?

Contaminated irrigation water is a well-known vehicle for transferring human pathogens onto crops (Alegbeleye et al., 2018), either by direct application onto the leaves or fruit, or indirectly, *via* splash from contaminated soil. Such pathways could also deliver colonised microplastics onto crops, either from microplastic-contaminated irrigation water or from microplastics being splashed up onto the plant from the soil (**Figure 1**). Once on the plant surface human pathogens are susceptible to a range of abiotic stresses in addition to any plant-specific defence responses (Zarkani and Schikora, 2021; Elpers et al., 2022). Enteric pathogens, such as *E. coli* and *Salmonella*, are poor at withstanding environmental factors such a UV irradiance and desiccation, and day length and temperature can affect the potential for epiphytic persistence on the leaf surface and consequently the concentration of viable or infectious cells remaining post cultivation (Alegbeleye et al., 2018). However, human pathogens in the plastisphere are offered some protection from environmental stressors by being part of a biofilm (Amaral-Zettler et al., 2020), which may facilitate survival on the surface of the plant and increase the potential for dissemination to other areas of the plant.

Recently, it has been demonstrated that nanoplastics and microplastics can be taken up and accumulated by plants, with apparent negligible effects on plant physiology (Azeem et al., 2021). Nanoplastics can enter plants through cracks or *via* the stomata, whereas due to their larger size, it is more likely that microplastics aggregate and are adsorbed onto the plant surface (Mateos-Cárdenas et al., 2021). Plant morphology and leaf topology will determine the strength of the interaction with microplastic particles, with the potential for microplastics to become trapped by trichomes and adhere to mucilage and exudates (Bi et al., 2020). The plastic polymer will also influence how strongly microplastics bind to leaves, with the charge on the plastic surface and the chemical bonds between the microplastic and the leaf surface making some microplastics extremely difficult to remove from crops such as lettuce, even after washing with water (He et al., 2023).

Plastic particles as large as 1 µm can enter the root tissue of rice seedlings and subsequently, driven by the pull of transpiration, be transported to the shoot (Liu et al., 2022). Although particles this size will not be colonised by bacteria, there is the potential for adherence of human viruses to microplastics of this size. Enteric viruses, such as rotavirus, which are commonly detected in treated effluents, surface waters, and irrigation water (Omatola and Olaniran, 2022), can bind to microplastics in the environment and remain infectious (Moresco et al., 2022). Human viruses preferentially bind to the biofilm of the plastisphere but depending on the pH and isoelectric point of the virus, are also capable of adhering to naked plastic particles through processes such as non-ionic forces (Moresco et al., 2021). Once associated with the plastisphere, human viruses (specifically, non-enveloped viruses) remain stable and seem to be protected from inactivation factors (Moresco et al., 2021), which could allow the transport through the plant vascular system and provide protection from changing environmental conditions and allow the pathogen to evade any plant-mediated defence response (Zarkani and Schikora, 2021).

Following the transfer of human bacterial pathogens such as *Salmonella enterica* to a leaf surface the expression of specific adhesive factors by the pathogen, e.g., fimbrial and nonfimbrial adhesins, can facilitate adhesion to the plant surface (Elpers et al., 2022), and may also be involved with adhesion to plastic surfaces (Yang et al., 2020). However, once microplastic particles have adhered to a plant surface, it is not known whether potential human pathogens in the plastisphere can then subsequently be transferred to the plant surface (either passively, by being shed from the plastisphere biofilm, or actively, by responding to chemotactic signals from the plant), or whether they would remain in the relative safety of the plastisphere. Either way, the plant microbiome, including phyllosphere communities, are likely to play an important role in any transfer of human pathogens from the plastisphere onto plants. In the short-term therefore, there is the potential for the co-pollutant risk of ingestion of both human pathogens and microplastics adhering to, or associated with, the surfaces of leaves or fruits eaten raw (**Figure 1**), whilst any toxins produced by specific foodborne pathogens in the plastisphere could remain even after cooking (Tavelli et al., 2022).

## Plastisphere - rhizosphere interactions

Contamination of agricultural soil with plastics is becoming ubiquitous, yet despite a growing knowledge of the diversity of soil plastisphere communities (Wang et al., 2022; Li et al., 2023) there remains little information on potential human pathogens colonising plastics in soil (Gkoutselis et al., 2021; Luo et al., 2022; Zhu et al., 2022). Plastics in agricultural soil can become contaminated by human pathogens through the introduction of livestock manure either as an organic fertiliser, or through direct deposition – similarly, open defecation can introduce human faeces into agricultural soil – or through irrigating soil with contaminated water. Binding to plastic surfaces in soil can facilitate the survival of human pathogens by providing a protective niche from abiotic stress, microbial grazing, and competition (Amaral-Zettler et al., 2020), and thus increases the potential for contact with roots or tubers.

The interaction of human pathogens with plant roots, and subsequent internalisation, has been well studied, with evidence for entry of bacterial pathogens through junctions in zones of lateral root emergence, following attraction by nutrients in root exudate (Zarkani and Schikora, 2021). Agricultural soils heavily contaminated with microplastics could allow potential human pathogens in the plastisphere continual access to the rhizosphere and developing roots over the course of the growing season, particularly if the plastisphere increases pathogen persistence in soil (**Figure 1**). Similarly, the opportunity for splash contamination of the aerial parts of crops would be greater if the inoculum potential of soil was amplified by the presence of human pathogens colonising microplastics. Bacterial pathogens (e.g., *Salmonella*) leaving the plastisphere in response to chemotactic signals in the rhizosphere could subsequently become pre-conditioned by plant exudate, with up-regulation of genes involved with virulence prior to internalisation in plant tissue (Jechalke et al., 2019). The potential for plastics to deliver human pathogens to the roots is unknown; however, in contrast to being in a biofilm, free-living bacterial enteric pathogens are likely to be more stressed, which often leads to higher expression of virulence (Zarkani and Schikora, 2021) with significant consequences for human health following ingestion.

## Conclusion

Although the potential for plastics in agricultural systems to be colonised by human pathogens is high, there is a lack of understanding of whether the interaction with crops is significant, and any more of a risk than free-living pathogens being introduced in water or soil. Therefore, in tandem with basic research on the potential risks of this novel delivery mechanism of pathogens to human receptors, there is an urgent need for both qualitative and quantitative risk assessments. Although the risk to human health from pathogens in the plastisphere has yet to be quantified, with limited evidence of virulence or pathogenicity of pathogens associated with the plastisphere (Beloe et al., 2022), the presence of anti-microbial resistance genes in the plastisphere is well known, particularly in soil (Zhu et al., 2022). Microbially diverse biofilm communities on plastics in soil increases the potential for multidrug resistance genes being transferred to human pathogens in the plastisphere, with more profound consequences for human health if transferred to ready to eat crops (Yang et al., 2022).

Providing the data to understand the risks of human pathogens in the plastisphere should now clearly be a priority as the concentrations of plastics in agricultural soils increases. Although the human health effects of ingesting microplastics at environmentally realistic concentrations is still being debated (Koelmans et al., 2022), the effects of ingesting microplastics colonised with human pathogens has never been quantified. Microplastics bound to ready to eat crops are difficult to remove by washing (He et al., 2023) and so could be acting as a vector for delivering human pathogens onto and into crops and subsequently provide a novel vehicle for human pathogens to enter the food chain.

## Data availability statement

The original contributions presented in the study are included in the article/supplementary material. Further inquiries can be directed to the corresponding author.

## Author contributions

RQ: Conceptualisation, Project administration, Writing - original draft, review & editing. CP: Figure design and drawing, Writing - review & editing. DJS: Writing -review & editing. JM: Writing -review & editing. DAS: Writing -review & editing. LW: Writing -review & editing. All authors contributed to the article and approved the submitted version.
